# Observations on Muco-Purulent Secretion of the Antrum Maxillare

**Published:** 1842-03

**Authors:** S. P. Hullihen

**Affiliations:** Wheeling, Va.


### ARTICLE VI.

### *Observations on Muco-purulent Secretion of the Antrum Maxillare.*

By S. P. Hullihen, Wheeling, Va.

This somewhat rare but painful disease, is doubtless, the result of a morbid secretion of the membrane lining the antrum maxillare. It is evidently constitutional in its nature, and probably of a scrofulous character.

Like most diseases of a constitutional diathesis, it appears to be always more or less mild or malignant, just in proportion as the constitutional taint, or predisposition is strong in the patient; and this must serve to explain the great difference in the severity of the disease in different patients.

Among the first indications of the disease is a slight inflammation in the pituitary membrane, and differing only from a common cold in being almost exclusively confined to one nostril. As the inflammation progresses, the nostril in consequence of a thickening of its membrane, closes; the tonsil of that side becomes enlarged; the eye always filled with tears, and a yellow watery discharge is almost continually flowing from the nose on the affected side. The length of time the disease is assuming this stage differs very widely in different cases.

The second stage is marked by a slightly fetid muco-purulent discharge from the nostril, which in severe cases is of a very thick consistence. The yellow watery discharge still continues, but generally not so frequent as the purulent form. The thickening of the pituitary membrane gradually subsides, the nostril opens, and in this situation the disease remains for a shorter or longer period, until the antrum which is scarcely ever suspected of being involved in the disease becomes filled with an altered secretion from its lining membrane.

If then the disease is mild in its form, a pain of a neuralgic character will most probably be felt over the eye for sometime.

Then a slight uneasiness in the antrum, and often a sensation of fullness on that side of the face. The discharge from the nostril will be sometimes watery, sometimes glairy, always very fetid, excoriating the nostril and blocking it up with troublesome incrustations. The disease is not unfrequently mistaken for ozœna, and may occasionally remain in the situation just described for several years before the walls of the antrum give way, and the true nature of the disease is revealed.

But where the disease is more malignant in its nature, in addition to the symptoms that accompany the milder form, a sensation of great weight or pressure will be felt in the antrum; after which a dull deep-seated pain supervenes; which is followed by an acute pain darting into the ear, through the temple and scalp, and over the eye in the direction of the frontal sinus. The eye waters incessantly; a thin sanious discharge is constantly passing from the nose. The cheek begins to project, the teeth to protrude from their sockets, the walls of the antrum at last give way, and a dark coloured secretion very thick and fetid of a slimy consistence, begins to escape through the opening. This generally takes place during the first year of the disease.

Now, in the first stage of this disease, it is evident from the thickening of the pituitary membrane, that the duct between the antrum and nose becomes closed, and judging from the state of this membrane in the second stage, it is likewise evident, that the duct re-opens before the antrum becomes filled with an altered secretion. The bursting then of this cavity from an accumulation of secretion within it, does not appear to proceed from the closure of this natural opening, but must be attributed to the character of the secretion itself. In the milder forms of the disease, the secretion being thin, it doubtless is discharged freely through the duct. But in the more malignant the secretion is of such a consistency as to prevent the possibility of its free escape through such an opening, its accumulation is therefore inevitable, and the bursting of the antrum is the consequence. The state of the teeth appears to have no agency whatever in producing this disease. Where it is most mild, the teeth are sometimes much decayed, and where it is most malignant, they are frequently sound. The apparent soundness of the teeth alone, however, is far from always indicating the true character of a disease in the antrum. A tooth may be apparently free from disease, and yet from the absorption of the gum and alveolus, the fangs may be so much exposed, and the nerve so much irritated from extreme degrees of heat and cold as to induce inflammation in its internal membrane, and finally suppuration, and an abscess may be the result.

*Treatment of Muco-purulent Secretion of the Antrum.*

The character of the disease now under consideration, requires both local and constitutional treatment.

On the milder form of the disease a perforation into the antrum, after the manner laid down in the first stage of abscess of this cavity, may be performed. But where the walls have already given way, and where the disease is malignant, the opening should be made with a reference to the state of the bony parieties of the antrum, which are generally more or less carious.

Where the bones are but slightly involved, the opening should be made to embrace if possible all the diseased portion; but where the caries is too extensive for this, the opening may be made of such dimensions as will enable the operator to examine fully the extent of the disease, and to remove with certainty all loose and diseased portions of bone.

After this the patient may be directed to syringe the antrum twice a day with a solution of the chloride of lime, which may be occasionally changed for a solution of the muriated tincture of iron, of lunar caustic or sulphate of zinc. The washes should be very weak at first, and gradually increased in strength until their effects are sensibly felt in the antrum for some time after each washing.

The patient will now be likewise ready for the constitutional treatment, and may be delivered over to a physician to be treated after the manner of other constitutional diseases of a similar nature. If this be neglected, the local treatment may it is true, often have the effect of gradually diminishing the discharge, and sometimes of checking it entirely for a time, until some derangement in the general health of the patient occurs, and then a renewed discharge from the antrum will most probably ensue. The condition of the general system must be first changed before a cure of this disease can with certainty be effected.

In the next number of the Journal, I propose to treat of another collection of fluids which occasionally occur in the antrum maxillare, differing in most particulars from both muco-purulent secretion and abscess of this cavity.

